# Retraction Note: Esoteric Connective Tissue Therapy for chronic low back pain to reduce pain, and improve functionality and general well-being compared with physiotherapy: study protocol for a randomised controlled trial

**DOI:** 10.1186/s13063-021-05154-3

**Published:** 2021-03-04

**Authors:** Christoph Schnelle, Steffen Messerschmidt, Eunice J. Minford, Kate Greenaway-Twist, Maxine Szramka, Marianna Masiorski, Michelle Sheldrake, Mark Jones

**Affiliations:** 1 School of Public Health, University of Queensland, Herston Road, Herston, QLD Australia; 2 Department of Surgery, Antrim Area Hospital, 45 Bush Rd, Antrim, BT41 2RL UK; 3 Queen’s University Belfast, University Rd, Belfast, BT7 1NN UK; 4 The Rheumatology Centre, Sydney and Nowra, Australia; 5 University of New South Wales, High Street, Kensington, NSW Australia; 6 Total Health From Inside Out Pty Ltd, Brisbane and Gold Coast, Australia; 7 Independent physiotherapist, practising in Goonellabah, Australia; 8 Independent psychologist, practising in Sunshine Coast, Australia

**Retraction Note: Trials (2017) 18:328**

**https://doi.org/10.1186/s13063-017-2055-8**

The authors are retracting this article as ethical approval has been withdrawn. The corresponding author, Christopher Schnelle, stated on behalf of all co-authors that they agree to this retraction.
